# Patient and disease characteristics associated with late tumour stage at presentation of cervical cancer in northwestern Tanzania

**DOI:** 10.1186/s12905-016-0285-7

**Published:** 2016-01-25

**Authors:** Ramadhani Mlange, Dismas Matovelo, Peter Rambau, Benson Kidenya

**Affiliations:** 1 Department of Obstetrics & Gynecology, Catholic University of Health & Allied Sciences, P.O. BOX 1464, Mwanza, Tanzania; 2 Department of Pathology, Catholic University of Health & Allied Sciences, P.O. BOX 1464, Mwanza, Tanzania; 3 Department of Biochemistry and Molecular Biology, Catholic University of Health & Allied Sciences, P.O. BOX 1464, Mwanza, Tanzania

**Keywords:** Cervical cancer, Late presentation, Advanced stage, Mwanza

## Abstract

**Background:**

About two thirds of patients with cervical cancer in Tanzania present with advanced tumor stage, leading to significant morbidity and mortality. We designed a study to determine the factors associated with the late tumour stage at presentation among patients with cervical cancer in Mwanza.

**Methods:**

This cross-sectional study recruited women at Bugando Medical Centre (BMC) with histologically confirmed cervical cancer from November 2013 to April 2014. Patients were recruited serially until the sample size was reached.

**Results:**

A total of 202 women with histologically confirmed cervical cancer were recruited. The mean age of the patients was 50.5 ± 13.3 years. The majority of patients (*n* = 129, 63.9 %) were diagnosed with late stage disease (IIB-IVB). Patients also presented with severe anemia (*n* = 78, 38.6 %), urinary tract infections (*n* = 74, 36.6 %), hydronephrosis (*n* = 43, 21.2 %), elevated serum creatinine levels (*n* = 33, 16.3 %), vesicovaginal fistula (VVF), (*n* = 13, 6.4 %), lung metastasis (*n* = 5, 2.4 %), metastasis to the urinary bladder (*n* = 4, 1.9 %), rectovaginal fistula (RVF) (*n* = 3, 1.4 %), liver metastasis (*n* = 2, 0.9 %) and hydroureter (*n* = 2, 0.9 %). In multivariate logistic regression, factors associated with late stage at presentation were attending to alternative health practitioners and lack of personal initiative to seek care to formal health facilities (OR 2.3; 95 % CI 1.2–4.2, *p* = 0.011 and OR 2.0; 95 % CI 1.0–3.8, *p* = 0.028) respectively.

**Conclusion:**

Communities should be sensitized to women’s empowerment, provide community education on early symptoms of cervical cancer, and the importance of early hospital attendance.

## Background

Cervical cancer is the fourth common cancer among women worldwide with nearly 529,000 incident cases and 275,000 deaths each year [1]. More than 85 % of these cases occur in developing countries due lack of implementation campaigns aimed at cancer prevention, such as screening for early detection and vaccination for Human Papillomavirus (HPV) infection [1]. The highest burden of cervical cancer-related deaths occur in developing countries and it is the leading cause of gynecological cancer-related deaths among women in Sub-Saharan Africa, Central America and South-Central Asia [2]. In Tanzania, approximately 3,000 new cervical cancer patients are seen at Ocean Road Cancer Institute per year and 47 % of these patients present with advanced stage disease [3]. At Bugando Medical Centre (BMC), 400 patients with cervical cancer are seen per annum [4].

Patients who present with advanced stage disease are more likely to develop complications such as anemia, ureteric obstruction with hydroureter and hydronephrosis, renal failure and urinary tract infection [5, 6]. Cervical cancer may present with abnormal vaginal discharge, contact vaginal bleeding, loin pain, changes in urine output, hematuria and increased serum creatinine, vesicovaginal fistula (VVF) and rectovaginal fistula (RVF) [7].

In developing countries, due to poor availability of health services for early screening and diagnosis, patients often present with advanced stage disease, which is associated with a poor prognosis and high mortality rate [8, 9]. Additionally, cultural, social and demographic factors may be responsible for patients’ presentation with late stage disease. A study from New Zealand reported that socioeconomic status and urban/rural residence had marginal effects [10]. Across all age groups, women in London with a lack of awareness and knowledge about cervical cancer were more likely to present with late stage disease [11]. In Morocco, an increased risk for a late stage was observed in women who were unmarried, lived in a rural area or more than 100 km from a diagnostic health center, did not have vaginal bleeding as their first symptom, were less than 50 years old, and were illiterate [12]. In a study conducted in Korea, other factors which contribute to the late diagnosis are inadequate knowledge of cervical cancer, lack of screening, and poor diagnostic procedure and treatment among health care provider [13].

At Bugando Medical Centre (BMC), in northwestern Tanzania, a previous study has shown that 47.3 % of patients with cervical cancer present with advanced-stage disease [4]. In Muhimbili National Hospital, Tanzania, more than 90 % of patients admitted were in an advanced disease (stage IIB-IV) and majority of the patients (50.6 %) were illiterate [14]. Despite these findings, it is not clear what factors contribute to late presentation of cervical cancer in East Africa. As a result, it has been difficult to develop targeted interventions to prevent late tumour stage at presentation. Findings from this study will provide baseline data to hospital authorities and policy makers to design effective and equitable interventions for prevention of late presentation to the hospital, which can ultimately minimize morbidity and mortality from cervical cancer.

## Methods

A cross-sectional study was conducted with patients diagnosed with cervical cancer from November 2013 to April 2014 at BMC, a referral, consultancy and teaching hospital for the Catholic University of Health & Allied Sciences (CUHAS) in Mwanza, Tanzania. The hospital serves about 13 million people from six regions of the Lake Zone, which are Kagera, Tabora, Shinyanga, Mara, Kigoma and Mwanza. The hospital has bed capacity for approximately 800 patients. It has also a well-equipped pathology laboratory staffed by two pathologists. About 3–4 women with suspected cancerous lesion of the cervix are seen weekly at the hospital for investigation and initial care before radical hysterectomy or radiotherapy. Approximately 400 cases of confirmed cervical cancer are seen yearly (BMC, unpubl).

Women who presented with suspected cervical cancer to BMC were identified and recruited serially until the sample size was reached. Written informed consent for participation in the study was obtained from each participants. Suspected cervical cancer patients were those with contact vaginal bleeding/postmenopausal bleeding, abnormal vaginal discharge, fecal/urine incontinence and pelvic/loin pain with or without visible cervical lesion on speculum examination. The final study population included women with histologically confirmed cervical cancer who were eligible for curative treatment. Patients were excluded if they were not competent to consent and did not have next of kin to provide informed consent, did not have confirmed cervical cancer, or who were treated with palliative intent for their cervical cancer.

Data were collected using pre-tested structured questionnaires. Information collected included clinical and pathologic staging, age, occupation, education, parity, marital status, residence, and results from laboratory and radiological investigations. The staging of carcinoma of the cervix was determined through bimanual examination under anesthesia with the support of chest X- ray and abdominal pelvic ultrasound. A cervical specimen from suspected cervical cancer patients was taken and kept in a 5 ml bottle containing 10 % formalin. The specimens were sent to the histopathology laboratory and were examined by a pathologist.

Venopuncture was performed where by a tourniquet 3–4 in. above the collection site was applied; the puncture site was cleaned by making a smooth circular pass over the site with the 70 % alcohol swab. When the skin dried a venopuncture was done with 5 cc syringe and 4mls of blood was drawn, 2mls of blood were distributed into plain and EDTA vacutainers and sent to the BMC laboratory for HIV serostatus, serum creatinine and hemoglobin level determination. In our hospital, a patient with confirmed cervical cancer clinical stage I-IIA are treated surgically (radical hysterectomy & pelvic lymphadenectomy) and the specimen is sent to the histology department for pathologic staging. Patients with advanced stage in both clinical and pathologic staging are referred for radiotherapy at Ocean Road Cancer Institute (ORCI). A mid-stream clean catch urine of approximately 5mls was sent to BMC laboratory for analysis of urinary tract infections (UTIs) by microscope.

Data were entered into Microsoft Excel and was analyzed using STATA® data analysis and statistical software package version 13 (StataCorp LP., 2013; College Station, TX, USA).

Continuous variables were summarized into means with standard deviations or medians with inter-quartile range, as appropriate. Categorical variables were summarized into frequencies and percentages. To determine factors related with late stage at presentation, univariate analysis was done followed by multivariate logistic regression. Odds ratios with 95 % confidence intervals were reported.

Ethical review and approval was obtained from the Joint Catholic University of Health & Allied sciences and Bugando Medical Centre’s research and publication committee. Written informed consent was requested and obtained from all participants in Swahili language and confidentiality was assured. Patient refusal to participate in this study did not jeopardize the quality of care that the patients received in the hospital.

## Results

A total number of 212 patients with clinical diagnosis of suspected cervical cancer were attended at BMC during the study period. Ten patients were excluded from the study; eight patients had no confirmed cancer and 2 patients did not consent to participate in the study. There were 202 patients with histologically confirmed cervical cancer who were included in the study.

### Characteristics of the study population

The mean age at presentation was 50.5 ± 13.3 years (range: 25–80 years). More than half 115 (56.9 %) of patients had no formal education. Of the patients with formal education, 39.1 % had primary level, 2.4 % had secondary level and 3 (1.4 %) had college level. Most of the patients (79.2 %) were coming from rural area, and majority 170 (84.1 %) were peasant farmers. The vast majority of patients (94 %, *n* = 190) had no medical insurance (see Table 1).

**Table 1:** Characteristics of the study population

Patient characteristic	*N* (%)
*Age in years*	
<40	45 (22.2)
40–59	101 (50.0)
≥60	56 (27.7)
*Marital status*	
Married	110 (54.4)
Single	9 (4.4)
Divorced	10 (4.9)
Separated	39 (19.3)
Windowed	34 (16.8)
*HIV status*	
Negative	150 (74.6)
Positive	44 (21.8)
Unknown	7 (4.4)
*Education level*	
Formal	87 (43.1)
None formal	115 (56.9)
*Occupation*	
Peasant	170 (84.1)
Petty trader	20 (9.9)
Business	2 (0.9)
Employed	5 (2.4)
Un-employed	5 (2.4)
*Residence*	
Urban	42 (20.7)
Rural	120 (79.2)
*Insurance*	
Insured	12 (5.9)
Un-insured	190 (94.0)
*Menopause*	
Yes	106 (52.4)
No	96 (47.5)
*Parity*	
<5	53 (26.2)
5–9	107 (52.9)
≥10	42 (20.7)

More than half of patients (54.4 %, *n* = 110) were living with their husband/sexual partners at the time of the study. Most women (73.6 %, *n* = 149) delivered more than five times in their lifetime. Forty-four (21.8 %) women were infected with HIV, and 106 (52.4 %) were menopausal (see Table 1).

### Clinical presentation and histological types

Contact vaginal bleeding was present in nearly all patients. Other symptoms included abnormal vaginal discharge (94 %) and pelvic pain (14.6 %). Squamous cell carcinoma was the most common tumour histology (see Table 2).

**Table 2:** Clinical presentation and histological types

Patient characteristics	*N* (%)
*Clinical presentation*	
Abnormal Vaginal discharge	190 (94.1)
Contact vaginal bleeding	201 (99.5)
Pelvic pain	29 (14.6)
*Stage*	
IA	3 (1.4)
IB	57 (28.2)
IIA	13 (6.4)
IIB	41 (20.4)
IIIA	32 (15.8)
IIIB	35 (17.3)
IVA	15 (7.4)
1VB	6 (2.9)
Early stages	73 (36.1)
Late stages	129 (63.9)
*Histopathology results*	
Squamous cell carcinoma	190 (94.1)
Adenocarcinoma	9 (4.4)
Clear cell carcinoma	3 (1.4)

### Prevalence of late stage cervical cancer among participants

Among 202 patients confirmed with cervical cancer, 73 (36.1 %) were found in an early tumour stage. The prevalence of late stage cervical cancer was 63.9 % (see Table 2).

### The pattern of complications among women with cervical cancer

Forty three patients (21.2 %) had hydronephrosis; of these, unilateral hydronephrosis was seen in 65.1 % and bilateral hydronephrosis in 34.9 % of the patients. Serum creatinine was elevated in 16.3 % of the patients. VVF was seen in 6.4 % of the patients, hydroureter (0.9 %) and liver metastasis (0.9 %). Other complications seen were severe anemia (38.6 %) and UTIs (36.6 %) (see Table 3).

**Table 3:** Pattern of complications among women with cervical cancer

Complications	*N* (%)
VVF	13 (6.4)
RVF	3 (1.4)
Lung Metastasis	5 (2.4)
Liver Metastasis	2 (0.9)
Urinary bladder metastasis	4 (1.9)
Hydroureter	2 (0.9)
Hydronephrosis	43 (21.2)
• Right • Left • Bilateral	12 (5.9) 16 (7.9) 15 (7.4)
Serum creatinine >139 mmol/l	33 (16.3)
Urinary tract infections	74 (36.6 )
Hemoglobin <7 g/dl	78 (38.6)

### Factors associated with late stage at presentation to the hospital

Univariate logistic regression was conducted to identify factors associated with a late tumour stage. Lack of formal education, lack of health insurance, attending to an alternative health practitioner, and lack of personal initiative to attend formal health care facility, and three or more pre-referral visits were statistically significant (*p* <0.05) (Table 4).

**Table 4:** Logistic regression model for factors associated with late stage at presentation

Variables	Late stage		Univariate		Multivariate	
	Yes	No	OR [95 % CI]	*p*-value	OR [95 % CI]	*p*-value
	*n* (%)	*n* (%)				
*Age in years*						
<40	29 (64.4)	16 (35.6)	1		1	
40–59	55 (54.5)	46 (45.5)	0.7 [0.3–1.4]	0.261	0.5 [0.3–1.2]	0.107
≥60	45 (80.4)	11 (19.6)	2.3 [0.9–5.5]	0.076	1.6 [1.1–3.8]	0.322
Marital status						
Living with a partner	66 (60.0)	44 (40.0 )	1			
Living alone	63 (68.5)	29 (31.5)	1.5 [0.8–2.6]	0.212		
*Education*						
Formal	47 (54.0)	40 (46.0)	1			
Non formal	82 (71.3)	33 (28.7)	2.1 [1.2–3.8]	0.012		
*Residence*						
Urban	24 (57.1)	18 (42.9)	1			
Rural	105 (65.6)	55 (34.4)	1.4 [0.7–2.9]	0.310		
*Insurance*						
Insured	4 (33.3)	8 (66.7)	1			
Un-insured	125 (65.8)	65 (34.2)	3.9 [1.1–13.3]	0.033		
*Menopausal*						
No	59 (61.5)	37 (38.5)	1			
Yes	70 (66.0)	36 (34.0)	1.2 [0.7–2.2]	0.499		
*Alternative-health practitioner*						
No	52 (53.1)	46 (46.9)	1		1	
Yes	77 (74.0)	27 (26.0)	2.5 [1.4–4.5]	0.002	2.3 [1.2–4.2]	0.011
*Health facility*						
Higher^a^	29 (64.4)	16 (35.6)	1			
Lower^b^	100 (63.7)	57 (36.3)	1.0 [0.5–1.9]	0.926		
*Health facility*						
Government	104 (63.0)	61 (37.0)	1			
Private	25 (67.6)	12 (32.4)	1.2 [0.6–2.6]	0.604		
*Personal initiatives*						
Yes	45 (51.7)	42 (48.3)	1		1	
No	84 (73.0)	31 (27.0)	2.5 [1.4–4.6]	0.002	2.0 [1.1–3.8]	0.028
*Number of pre-referral visit*						
≤2	39 (54.2)	33 (45.8)	1			
≥3	90 (69.2)	40 (30.8)	1.9 [1.1–3.5]	0.034		
*HIV status*						
Negative	95 (63.3)	55 (36.7)	1			
Positive	28 (63.6)	16 (36.4)	1.0 [0.5–2.0]	0.971		

^a^Hospital

^b^Health center and dispensary

In multivariate logistic regression; only attending to traditional health practitioners and lack of personal initiative to attend health care facility were associated with a late tumour stage at presentation (OR = 2.3 [95 % CI 1.2–4.2], *p* = 0.011) and OR = 2.0 [95 % CI 1.0–3.8], *p* = 0.028) respectively.

## Discussion

Despite efforts to curb the morbidity and mortality attributed to cervical cancer; deaths and complications are the most burdensome in developing countries, including Tanzania. The prevalence of late tumour stage at presentation to the hospital in our study was 63.9 % which is lower than those seen in studies from Zaria Northern Nigeria (98 %), Muhimbili National hospital Tanzania (90 %), Nepal (80.9 %) and India (80 %) [8, 14–16], but higher than reported in a Morocco study (54.5 %) [12]. The higher prevalence found in Nigeria, Muhimbili National Hospital, Nepal, and India could be because their study sites are located within oncology centers where patients are referred for radio-chemotherapy; there might be a delay in referrals causing patients to arrive to these centers in advanced stage. The difference can also be explained by geographical variations and socioeconomic status, which hinder the accessibility to health care facilities and poor health seeking behaviors, when compared to our study population. The Morocco study likely reported lower rates because of differences in classification of advance tumor stage; their study classified advanced tumour stage as stage III-IVB, whereas this study categorized advanced tumour stage as IIB –IVB.

Anemia and hydronephrosis were the most common complications seen in this study. These findings were similar to studies done in Uganda and Minnesota [6, 17]. In this study; 65.1 % of the sample had unilateral hydronephrosis, and elevated serum creatinine was found in 16.3 % of all patients. These findings are similar to a study done in Sub-Saharan Africa where most of their patients 56 % had unilateral hydronephrosis and 15.1 % of patients had elevated serum creatinine [18]. Our study found that elevated serum creatinine (16.3 %) was less than what was previously reported in Uganda (71.4 %) [6]. The low occurrence of elevated serum creatinine in our study is due to the inclusion of all patients with cervical cancer while for a study in Uganda only patients with hydronephrosis were included.

In this study VVF was found in 6.4 % of all patients with cervical cancer which was similar to studies done in Minnesota (7 %) and Uganda (7.4 %) respectively [6, 17].

Attending to alternative health care practitioners and lack of personal initiative for early attendance to health care facility were the main factors associated with late tumour stage at presentation. These findings were similar to a study done in Uganda [19]. In our study age and lack of health insurance were not related with a late stage at presentation, contrary findings from Florida and Sudan [20, 21]. The difference could be attributed by cultural differences, health seeking behavior and health systems. In our setting most women have no health insurance and have never been screened for cervical cancer in their lifetime, as compared to developed countries where most women have insurance and are screened regularly [20]. All factors subjected in the univariate analysis and later multivariate analysis may have a causal link to each other, although no linkage is seen in multivariate analysis. The pre-referral visit, lack of personal initiative to seek care, and opting for alternative health practitioners could be attributed either to an inability of women in the community to make their own decision regarding their health, where either their husbands or close relatives decide for them, or may be associated with a low level of education.

This study has several limitations. Investigations such as intravenous pyelogram (IVP), magnetic Resonance imaging (MRI), cystoscopy and urine for culture and sensitivity were not performed due to limited funds. However, ultrasound has shown a high sensitivity for detecting hydronephrosis when compared to IVP [22]. The frequency of stage IIB, bladder metastasis and urinary tract infections may have been underestimated as MRI, cystoscopy and urine for culture and sensitivity were not performed. Since this was a hospital-based study, there is possibility of underestimating the understanding and belief within rural communities with respect to cervical cancer.

## Conclusion

Communities should be sensitized on women empowerment to be actively engaged in health care decision-making, provide health education on early symptoms of cervical cancer and the importance of early hospital attendance when symptoms arise.

## Abbreviations

BMC: Bugando Medical Centre
CUHAS: Catholic University of Health & Allied Sciences
IVP: intravenous pyelogram
MRI: magnetic resonance imaging
RVF: rectovaginal fistula
UTIs: urinary tract infections
VVF: vesicovaginal fistula
